# PhenoRob-P: An autonomous robotic system for high-throughput phenotyping of potted plants

**DOI:** 10.1016/j.plaphe.2026.100232

**Published:** 2026-06-06

**Authors:** Yang Shao, Yan He, Yakun Fang, Zhengda Li, Ruifang Zhai, Wanneng Yang, Peng Song

**Affiliations:** a National Key Laboratory of Crop Genetic Improvement, National Center of Plant Gene Research, Hubei Hongshan Laboratory, Huazhong Agricultural University, Wuhan, 430070, China; b Wuhan X-Agriculture Intelligent Technology Co., Ltd., Wuhan, 430014, China

**Keywords:** Phenotyping robot, High-throughput phenotyping, Multi-sensor fusion navigation, Adaptive phenotyping data acquisition, Cloud-edge collaboration

## Abstract

High-throughput phenotyping is essential for resolving genotype-by-environment interactions and accelerating crop breeding. In greenhouse potted-plant systems, narrow aisles, global navigation satellite system (GNSS)-denied operation, variable pot layouts, and plant-level data traceability constrain repeatable automated phenotyping. This study presents PhenoRob-P, a modular autonomous robotic system designed for potted crops in structured facility environments. The system integrates a compact two-wheel differential chassis, a LiDAR–vision fusion framework for row-level navigation, pot-level target identification and local alignment, a six-degree-of-freedom robotic arm with inverse-kinematics-based real-time pose compensation for repeatable multi-view close-range imaging, and a three-tier User–Cloud–Robot platform for task scheduling, remote monitoring, and closed-loop data management. Greenhouse validation showed throughputs of 520 pots/h in continuous scanning mode and 187 pots/h in multi-view fine inspection mode. At travel speeds of 0.2–0.3 m/s, mean terminal positioning errors remained within 30 mm, and approximately 87% of lateral and longitudinal errors fell within ±30 mm. Biological validation demonstrated time-resolved stress phenotyping in wheat, with color indices capturing drought progression and rewatering recovery. For maize, multi-view three-dimensional reconstruction estimated plant height and stem diameter with R^2^ values of 0.940 and 0.845, respectively, relative to manual measurements. These results show that PhenoRob-P provides an integrated perception-localization-acquisition-analysis workflow for high-throughput, traceable, and time-resolved phenotyping of potted crops.

## 1 Introduction

High-throughput phenotyping is essential for linking genotype to phenotype and has become a key enabling technology for modern crop breeding, functional genomics, and stress biology [[1], [2], [3], [4]]. In controlled-environment agriculture, this need is particularly pronounced because greenhouse and other facility-based production systems support year-round cultivation, precise environmental regulation, and repeated observation of the same plants across developmental stages [[5], [6], [7], [8]]. The value of such systems for breeding, however, depends not only on environmental controllability but also on whether phenotypic data can be acquired accurately, repeatedly, and at scale throughout the crop life cycle [9].

Tracking crop phenotypes across the full growth cycle remains particularly challenging in potted crop production systems. Compared with open-field conditions, these environments are commonly characterized by narrow aisles, complex facility structures, dense cultivation layouts, and frequent occlusion from surrounding infrastructure [[10], [11], [12]]. In addition, greenhouse and protected cultivation settings are typically GNSS-denied, which complicates reliable autonomous navigation over long operational periods [[11], [12], [13]]. For mobile phenotyping robots, these constraints create requirements that extend beyond basic collision-free movement. In practice, robust operation depends on repeatable localization, reliable plant-level identification, and stable sensor-to-plant geometry, all of which are necessary for generating comparable time-series phenotypic measurements across plants, positions, and growth stages [[14], [15], [16]].

A wide range of phenotyping platforms has been developed to improve throughput and reduce the labor cost and subjectivity of manual sampling, including conveyor-based systems, gantry systems, UAVs, and ground robots [1,4,14,17]. Stationary platforms provide high sensor stability and payload capacity, which are advantageous for precise imaging and multimodal sensing, but their infrastructure dependence, fixed viewpoints, and deployment cost may limit flexibility and scalability [1,18,19]. UAV-based systems enable rapid coverage in open-field settings, yet their payload, endurance, and viewpoint stability remain restrictive when close-range sensing or high spatial consistency is required [[20], [21], [22]].

Ground robots offer an effective compromise among mobility, endurance, payload, and sensing proximity, and have therefore become an important direction in agricultural phenotyping [23]. In field scenarios, representative systems such as PhenoRob-F, TERRA-MEPP, and TerraSentia have demonstrated the feasibility of autonomous trait acquisition across different crop types and growth stages [[24], [25], [26]]. In facility agriculture, robotic systems such as G-ROBOT and PATHoBot have further shown the potential of robotic monitoring in greenhouse environments [[27], [28], [29]]. Representative systems have demonstrated autonomous trait acquisition in both field and greenhouse settings, confirming the value of embodied sensing for crop monitoring.

Despite these advances, an important gap remains for autonomous phenotyping in potted plant production scenarios. Many existing systems are designed primarily for row following, rail-guided operation, or region-level monitoring, rather than for repeated plant-specific acquisition at the pot level [11,13]. Standard map-based navigation frameworks can support localization and path tracking, but they do not by themselves ensure reliable association between phenotypic data and an individual plant, nor do they guarantee repeatable sensor pose relative to the target during multi-view acquisition [30]. These requirements are particularly important for phenotypic tracking across the full crop growth cycle, because navigational deviation, target ambiguity, and viewpoint inconsistency can directly compromise the comparability of phenotypic observations across time [30,31]. In addition, although marker-free and fully vision-driven systems represent an important long-term direction, practical breeding workflows still often require plant- or pot-level identity management to ensure traceable sample organization and continuous phenotypic tracking across the full crop growth cycle [[31], [32], [33]]. This creates a need for a deployable intermediate solution that balances automation, robustness, and data traceability in real production environments [32,33].

At the same time, the operational scope of mobile phenotyping robots in greenhouse systems should be defined realistically. Dense commercial canopies with severe overlap, multilayer cultivation systems, and large variations in crop height remain challenging when reliable target separation and repeatable close-range observation are required [11,34,35]. Under such conditions, a single-plane 2D LiDAR cannot represent the full three-dimensional complexity of the scene [36,37]. In the present study, the 2D LiDAR is used primarily for global localization and row-level navigation, rather than for detailed structural reconstruction. This sensing configuration reflects a practical task-oriented trade-off for potted production environments, where reliable row traversal, pot-level target identification, and repeatable proximal acquisition are the primary operational requirements.

To address these needs, this study presents PhenoRob-P, an autonomous phenotyping robot system designed for potted plant production environments. The originality of this work lies primarily in a task-oriented system integration for autonomous phenotyping rather than in proposing a completely new generic robotics framework. Specifically, the system combines narrow-aisle mobile operation, LiDAR–vision collaborative localization, pot-level identification and local alignment, robotic-arm-based multi-view acquisition, and cloud-enabled task and data management into a unified workflow for plant-specific and time-resolved phenotyping. By doing so, it aims to solve three practical problems that are not fully addressed by standard off-the-shelf navigation packages alone: reliable operation in GNSS-denied narrow-row facility environments, robust association between phenotypic data and individual potted plants, and improved geometric consistency during repeated multi-angle acquisition.

The main contributions of this study are as follows.

(1) We developed a modular intelligent phenotyping robot architecture for potted plant environments, integrating autonomous navigation, crop-container recognition and positioning, robotic-arm-based acquisition, and cloud-based task and data management to support automated monitoring across the crop growth cycle.
(2) We established a LiDAR–vision collaborative perception and pot-positioning workflow for facility environments, enabling row-level navigation together with pot-level target identification and local alignment for repeatable plant-specific data acquisition.
(3) We implemented an adaptive multi-view phenotyping module based on a 6-DoF robotic arm and pose-compensation strategy, which improves sensor-to-plant geometric consistency during close-range imaging and supports extensible multimodal phenotyping.
(4) Through greenhouse experiments on navigation performance and biological applications, we validated the practicality of the proposed system for high-throughput monitoring, dynamic stress phenotyping, and structural trait extraction in potted crop production scenarios.

## 2 Robot system architecture

### 2.1 System overview

**Fig. 1:**
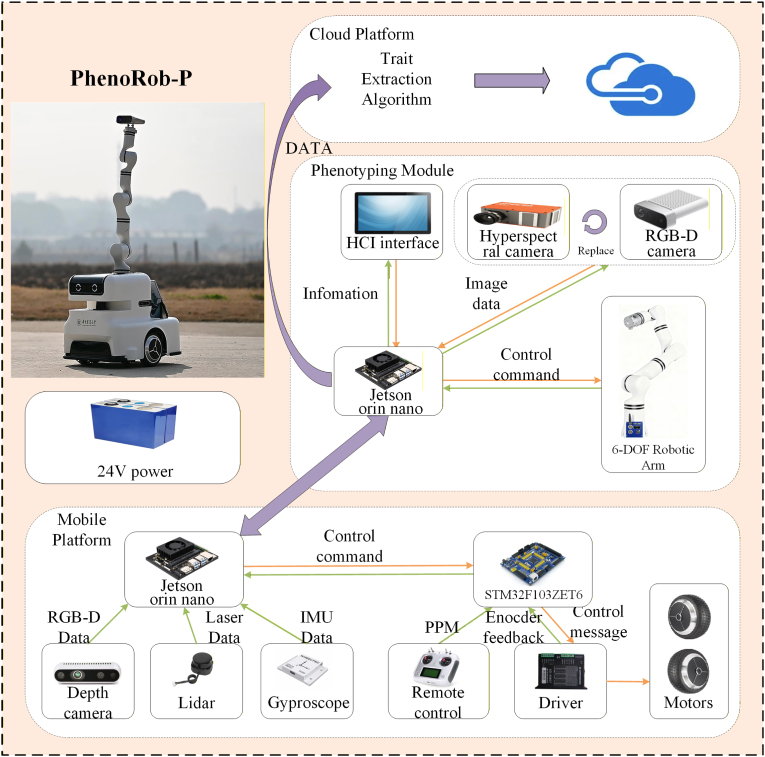
System architecture diagram of the phenotyping robot in controlled environments.

To capture the dynamic phenotypic changes of crops throughout their entire growth cycle while addressing the varying analytical demands across different scenarios, this study presents the design of the PhenoRob-P phenotyping robot system. The system adopts an in-situ sensor-to-plant data collection model, enabling non-destructive and high-throughput phenotyping. As shown in Fig. 1, the system comprises three core components: an autonomous mobile chassis, an adaptive robotic arm-based phenotyping module, and a cloud-based management platform. The autonomous mobile chassis achieves real-time perception and localization of the surrounding facility environment and individual crop containers by fusing data from LiDAR, Inertial Measurement Units (IMU), and depth cameras. This multi-sensor fusion enables the robot to autonomously plan paths based on phenotyping tasks, precisely identifying and approaching specific targets to execute data collection. The adaptive robotic arm acquisition platform relies on a multi-degree-of-freedom (DoF) robotic arm equipped with interchangeable end-effectors for sensors. This design facilitates multi-angle and multi-type stable phenotypic acquisition. A human–computer interaction interface (HCI) allows operators to freely configure specific target areas and angles for precise data collection. Furthermore, based on spatial positioning provided by depth cameras, the robotic arm dynamically adjusts its relative pose to ensure the stability and consistency of the acquired data. The cloud-based data management and analysis platform is deployed in a high-performance computing service center. This platform enables edge-cloud collaboration, leveraging substantial computational power for the immediate processing and analysis of phenotypic traits. Additionally, it supports task scheduling and remote monitoring of the robot system.

### 2.2 Design of the autonomous mobile robot platform

#### 2.2.1 Design of the robot hardware structure

**Fig. 2:**
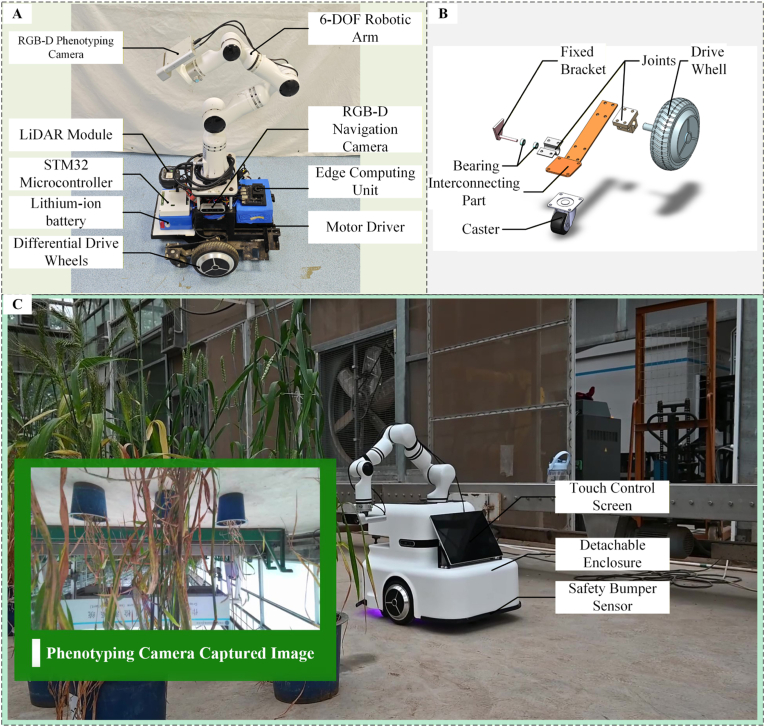
Hardware diagram of the phenotyping robot System. (A) Hardware components of the robot; (B) dual-wheel differential suspension Structure; (C) operational scene of the robot with protective casing.

To ensure the robot's flexibility and precise positioning within constrained facility environments, this study developed a compact mobile platform (Fig. 2A). Considering typical greenhouse layouts and the majority of standard breeding containers, the platform dimensions were established at 65 cm (L) × 50 cm (W) × 30 cm (H), with a wheel track of 44 cm. The platform employs a two-wheel differential drive mechanism, where steering is achieved through the differential speed control of two independent motors. This design enables in-place pivot turns with a zero turning radius, ensuring flexible maneuvering within narrow aisles (<60 cm) and unhindered mobility across constrained greenhouse environments. The high-level system control is hosted on a Jetson Orin Nano edge computing unit. This unit is primarily responsible for navigation, visual container localization, and robotic arm control. Low-level motion control is managed by an STM32 processor. This controller communicates with the edge computing unit via USB to upload real-time odometry data and receive navigation commands, which are subsequently distributed to the motor drivers via a CAN bus. For navigation-related perception, the robot integrates a 2D LiDAR for global localization and obstacle avoidance, an onboard IMU for real-time motion-state estimation, and a side-view RGB-D camera (RealSense D435i) for pot detection, ID recognition, and local spatial positioning of target plants. These sensors are therefore mainly used to support autonomous movement, target association, and local alignment rather than direct phenotypic trait extraction. By contrast, the phenotyping module is mounted on a six-degree-of-freedom robotic arm and uses an end-effector sensor for close-range crop data acquisition. In the current implementation, this end-effector is an RGB-D phenotyping camera (Azure Kinect DK), which is used to acquire high-resolution multi-view phenotypic data for downstream trait extraction. Owing to the modular quick-release interface, the end-effector sensor can also be replaced by other sensing devices, such as near-infrared or hyperspectral cameras, according to specific phenotyping requirements. For clarity, the main hardware configuration and representative system-level performance indicators of PhenoRob-P are summarized in Table 1.

**Table 1:** System specifications and representative performance metrics of PhenoRob-P.

Category	Item	Value
System specification	Mobile platform size	65 cm × 50 cm × 30 cm
	Chassis configuration	Two-wheel differential-drive mobile platform
	Computing hardware	Jetson Orin Nano (high-level control) + STM32 (low-level control)
	Navigation sensors	2D LiDAR + IMU + side-view RGB-D camera (RealSense D435i)
	Manipulator specification	6-DoF robotic arm, 5 kg rated payload, 610 mm working radius, ±2 mm repeatability
Representative performance	Tested travel speed	0.2–0.3 m/s
	Navigation accuracy	Mean terminal positioning error within approximately 30 mm
	Scanning throughput	520 pots/h (continuous scanning mode)
	Multi-view phenotyping throughput	187 pots/h (multi-view fine inspection mode)

To enhance motion control precision, suspension mechanisms were installed on both drive wheels (Fig. 2B). The swing-arm suspension design ensures continuous ground contact, thereby minimizing motion errors caused by tire slippage and low friction. To support extended operation, the chassis is equipped with a 24V, 30Ah Lithium Iron Phosphate battery. Field tests demonstrated that the platform operates continuously for 4-6 h in greenhouse environments, ensuring sufficient power for sustained, high-throughput phenotyping tasks. Fig. 2C depicts the robot operating in a real-world scenario with its protective casing.

#### 2.2.2 Robot vision and LiDAR fusion navigation

Mobile platforms relying on 2D LiDAR navigation offer substantial flexibility. However, they are incapable of precisely localizing individual pots using laser scanning alone [36]. As a result, collected data are often restricted to regional-level information, precluding the direct association of phenotypic traits with specific pot identifiers. These limitations significantly constrain the operational efficiency of current devices designed for potted plants.

**Fig. 3:**
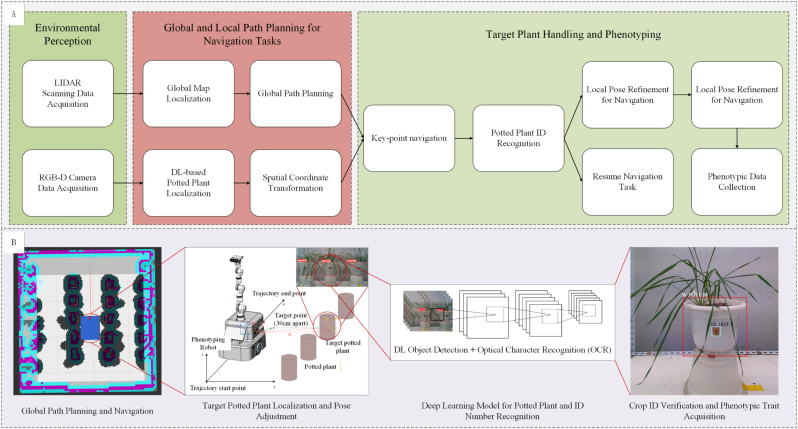
Integrated Navigation Architecture of the Phenotyping Robot. (A) LiDAR-Vision Fusion-Based Navigation Architecture; (B)Navigation pose correction, potted plant localization, and ID recognition workflow.

Accordingly, we propose an improved navigation scheme that fuses LiDAR and vision for the PhenoRob-P. As shown in Fig. 3A, the robot utilizes an Oradar M500 LiDAR for autonomous navigation. Simultaneously, a side-view RGB-D navigation camera (RealSense D435i) is used for target-pot detection, OCR-based ID recognition, and local 3D pose estimation, thereby supporting target association and local alignment prior to phenotypic acquisition. Building on this identification, the system further resolves the 3D spatial pose of each pot, thereby enabling precise perception of the target's position and orientation.

Fig. 3B details the robot's navigation workflow. Initially, a global occupancy grid map of the greenhouse is constructed using the *slam_toolbox* SLAM algorithm. Key start and end points for the pot rows are annotated on this map via software. Each keypoint contains coordinate information (*x*, *y*, *θ*) within the map reference frame. These point pairs define the start and end of the robot's operation paths, serving as the basis for global path planning and local cruising. To initiate a phenotyping mission, the user issues commands via the software interface, specifying the target pot identifier, the operation start point, and the data acquisition mode (scanning or multi-angle). Upon receiving these instructions, the robot navigates to the designated start point to commence the task. Once at the start point, the robot activates its side-view depth camera and traverses toward the endpoint at a constant velocity along the preset path. During motion, the system detects the spatial positions of pots in real-time. A deep learning model is employed to precisely localize detected targets, obtaining their 3D coordinates relative to the depth camera. Subsequently, the image is cropped within the localized target area to isolate the pot identifier region, which is then decoded using an OCR algorithm. The recognition result is matched against the target identifier in the task database. Once a match is confirmed, the robot triggers the subsequent phenotypic data acquisition sequence. As shown in the figure, the depth camera frame is denoted as $F_{C}\left(x_{c},y_{c},z_{c}\right)$ and the robot base frame as $F_{r}\left(x_{r},y_{r},z_{r}\right)$. The deep-learning localization module outputs the homogeneous coordinate of the pot center in $F_{C}$, expressed as:

(1) $$\begin{array}{c} p_{c}=\left[\begin{array}{c} x_{p}\\ y_{p}\\ \begin{array}{c} z_{p}\\ 1 \end{array} \end{array}\right] \end{array}$$

Because $F_{C}$ and $F_{r}$ are rigidly connected, their relationship can be described by a constant coordinate transformation matrix:

(2) $$\begin{array}{c} T_{c}^{r}=\left[\begin{array}{cc} R_{\text{rc}} & t_{\text{rc}}\\ 0 & 1 \end{array}\right]\in \text{SE} \end{array}$$

The matrix $R_{\text{rc}}\in R^{3\times 3}$ represents the rotation from the camera frame to the robot base frame, and $t_{\text{rc}}\in R^{3\times 1}$ denotes the corresponding translation vector.

According to the homogeneous transformation relation, the three-dimensional position of the pot center in the robot base frame, $p_{r}$, can be written as:

(3) $$\begin{array}{c} \left[\begin{array}{c} \begin{array}{c} x_{r}\\ y_{r} \end{array}\\ z_{r}\\ 1 \end{array}\right]=T_{c}^{r}\left[\begin{array}{c} \begin{array}{c} x_{p}\\ y_{p} \end{array}\\ z_{p}\\ 1 \end{array}\right] \end{array}$$

Which can be expressed in compact form as

(4) $$\begin{array}{c} p_{r}=T_{c}^{r}p_{c} \end{array}$$

Upon the completion of localization, the region containing the pot identifier is extracted via image cropping. Subsequently, an Optical Character Recognition (OCR) algorithm is employed to decipher the identifier, and the recognition result is matched against target identifiers within the working database. When the detected pot identifier aligns with a pending target, a custom local pot alignment algorithm is utilized to ensure the crop is positioned within the manipulable workspace of the robotic arm for phenotypic imaging. Specifically, based on the acquired coordinates $\left(P_{rx},P_{ry}\right)$ of the target pot, this algorithm calculates the distance and deviation angle relative to a waypoint located 30 cm directly in front of the pot. A PID controller drives the robot to this position in real time. Once aligned, the phenotyping module acquires the image data of the target plant. If the pot number has already been processed or does not match the task list, the robot bypasses it and continues navigating along the row. This workflow enables precise matching between image data and pot identifiers, improves operational efficiency, and ensures reliable data association.

### 2.3 Adaptive robotic arm phenotyping acquisition platform

**Fig. 4:**
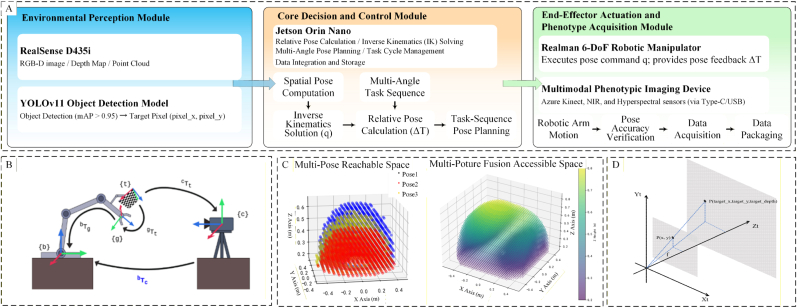
Architecture of the Adaptive Robotic Arm Phenotyping Platform. (A) System workflow of the adaptive phenotyping platform; (B) Hand-Eye calibration for the multi-DOF robotic arm (Eye-to-Hand configuration); (C) Reachable workspace expansion via inverse kinematics solving; (D) Dynamic pose correction mechanism.

In potted plant production scenarios, existing mobile phenotyping platforms often fail to maintain dynamic pose alignment between the crop and the sensing unit. This misalignment leads to geometric inconsistencies and measurement errors during multi-view data acquisition, which in turn reduces the accuracy and repeatability of phenotypic trait extraction. To address this limitation, we developed an adaptive robotic-arm module that integrates a multi-DOF manipulator with modular end-effectors. This module was designed to support fine phenotyping from selectable multiple viewpoints around the plant, while maintaining a stable relative pose between the sensor and the crop throughout the acquisition process. A real-time pose compensation algorithm based on analytical inverse kinematics is employed to maintain stable and consistent sensor-to-crop alignment throughout the acquisition process. Therefore, the adaptive robotic-arm platform not only enables customizable multi-angle phenotyping acquisition according to experimental requirements, but also improves the consistency and repeatability of phenotypic data collected across different observations. This design supports multi-view phenotyping and allows flexible replacement of sensing modules according to crop type or stress condition, enabling scalable multimodal perception (e.g., RGB, NIR, and hyperspectral imaging). By extending observation from a single side view to multiple viewpoints, the platform also helps reduce information loss caused by occlusion or canopy overlap, thereby providing more complete structural data for subsequent trait analysis. The overall architecture of the adaptive robotic-arm phenotyping platform, including the system workflow, hand–eye calibration, reachable workspace expansion, and dynamic pose correction mechanism, is illustrated in Fig. 4.

The adaptive robotic-arm module consists of a Realman RM65-B 6-DOF manipulator, a modular end-effector for phenotyping acquisition, and a control algorithm based on inverse kinematics. The manipulator communicates via a serial interface with the robot's edge-computing unit (Jetson Orin Nano), which uses a RealSense D435i depth camera and the YOLOv11 detection model to identify and localize target pots in real time. The three-dimensional coordinates (x, y, z) of the target pot are transmitted to the arm controller, which computes the corresponding joint angles through the analytical inverse kinematics solution, enabling precise control of the manipulator's motion. Based on the target position, the sensor can then be guided to a predefined or task-dependent sequence of viewpoints for multi-view phenotyping acquisition.

To ensure pose stability during data acquisition, the robotic arm controller continuously computes the pre-acquisition pose deviation and dynamically adjusts the end-effector position to compensate for motion-induced errors. Before each image capture, pose consistency is verified by the dynamic compensation module, and acquisition is triggered only when the angular deviation is below 0.5° and the translational deviation is below 2 mm. To avoid computational delays caused by time-sensitive requirements, a simplified inverse kinematics solution strategy was adopted. By applying suitable matrix transformations, the problem is converted into efficient numerical calculations, substantially reducing computational cost. Specifically, the system generates the corresponding homogeneous transformation matrix from the target coordinates and, combined with the known joint angles of the robotic arm, derives the joint angle solutions via matrix operations. Assuming each joint of the robotic arm has a homogeneous transformation matrix Tii−1:

(5) $$\begin{array}{c} {\;}_{i}^{i-1}T=\left[\begin{array}{cccc} c\theta_{i} & -s\theta_{i} & 0 & a_{i-1}\\ s\theta_{i}c\alpha_{i-1} & c\theta_{i}c\alpha_{i-1} & -s\alpha_{i-1} & -s\alpha_{i-1}d_{i}\\ s\theta_{i}s\alpha_{i-1} & c\theta_{i}s\alpha_{i-1} & c\alpha_{i-1} & c\alpha_{i-1}d_{i}\\ 0 & 0 & 0 & 1 \end{array}\right] \end{array}$$

The overall transformation matrix of the system can be expressed as ${\;}_{0}^{6}T{=}_{1}^{0}T\cdot_{2}^{1}T\cdot_{3}^{2}T\cdot_{4}^{3}T\cdot_{5}^{4}T\cdot_{6}^{5}T$, For inverse kinematics, the target coordinates and pose are known, allowing determination of a unique end-effector pose:

(6) T60=[nxoxaxpxnyoyaypynzozazpz0001]

To compute the joint angles $\left[\theta_{1},\theta_{2},\theta_{3},\theta_{4},\theta_{5},\theta_{6}\right]$ , we solve the relation

(7) T60=[nxoxaxpxnyoyaypynzozazpz0001]=10T·21T·32T·43T·54T·65T

Substituting all Tii−1 matrices from above and equating corresponding elements yields a system of equations for the joint angles. However, directly solving this system is too time-consuming for high-speed robotic motion. To simplify the computation, suitable left- or right-multiplications with inverse matrices are applied, reducing the system to a simplified form. By comparing corresponding elements of the transformed matrices, the equations for the joint angles can be obtained:

(8) T10−1·06T=21T·32T·43T·54T·65T

Thus, the combined system of equations becomes:

(9) $$\begin{array}{c} \left\{ \begin{array}{c} a_{y}c\theta_{1}-o_{x}s\theta_{1}=-s\theta_{4}s\theta_{5}\\ p_{y}c\theta_{1}-p_{x}s\theta_{1}=-144s\theta_{4}s\theta_{5}\\ \begin{array}{c} g\left(\theta_{1}\right)=107520s\theta_{3}+109636\\ \begin{array}{c} p_{z}-144a_{z}-240.5=256s\theta_{2}-210c\left(\theta_{2}+\theta_{3}\right)\\ -c\theta_{5}=a_{z}c\theta_{2}c\theta_{3}-a_{z}s\theta_{2}s\theta_{3}-a_{x}c\theta_{1}c\theta_{2}s\theta_{3}\\ \begin{array}{c} -a_{x}c\theta_{1}c\theta_{3}s\theta_{2}-a_{y}c\theta_{2}s\theta_{1}s\theta_{3}-a_{y}c\theta_{3}s\theta_{1}s\theta_{2}\\ -s\left(\theta_{2}+\theta_{3}\right)s\theta_{4}=o_{z}c\theta_{6}+n_{z}s\theta_{6} \end{array} \end{array} \end{array} \end{array}\right. \end{array}$$

This step is critical for high-speed robotic motion control. By employing a fast numerical solution, computation time is minimized, satisfying the requirements for real-time control.

In this system, the Azure Kinect DK depth camera is mounted as the end-effector sensor and connected to the onboard computer through a Type-C interface, enabling high-resolution phenotypic image acquisition and real-time data transfer. The modular end-effector design allows replacement with a near-infrared camera for monitoring physiological stress responses or with a hyperspectral imager for assessing biochemical traits, ensuring compatibility with diverse phenotypic parameters. During the phenotyping workflow, the side-view RealSense D435i depth camera first performs continuous scanning of the operational area. Once a target pot enters the center of the field of view and is confirmed as a valid target by a YOLOv11 model with mAP exceeding 0.90, the mobile chassis adjusts its pose and stops. The onboard computer then initiates the robotic arm's initialization sequence: it extracts the pot's center pixel coordinates (pixel_x, pixel_y) from the RGB-D data and computes the corresponding depth value (target_depth) by aligning the point cloud with the color image, thereby generating the initial 3D point for subsequent motion planning.

Subsequently, the system employs homogeneous transformation matrices—including camera intrinsic calibration and robot extrinsic registration—to map the image coordinates to the robot base coordinate system, generating the relative pose transformation matrix of the potted plant. For multi-view fine phenotyping, the robotic arm then guides the sensor through a predefined or experimentally adjustable sequence of viewpoints, such as front, top, and left/right side views, to capture images from multiple observation angles around the plant. To achieve standardized multi-angle acquisition, the robotic arm follows a predefined sequence of orientations for end-effector adjustment and verifies pose consistency before each capture, with thresholds of angular deviation <0.5° and translational deviation <2 mm. During this process, the end-effector scanning speed was set to 0.2 m/s, while the camera settings and chassis travel speed remained unchanged during row traversal. Compared with continuous side-view scanning, this mode provides more complete geometric and morphological information and improves the reliability of phenotypic observation under partially occluded conditions. This dynamic pose compensation strategy significantly improves the geometric consistency and repeatability of the acquired phenotypic data.

### 2.4 Cloud-based data management and analysis platform

**Fig. 5:**
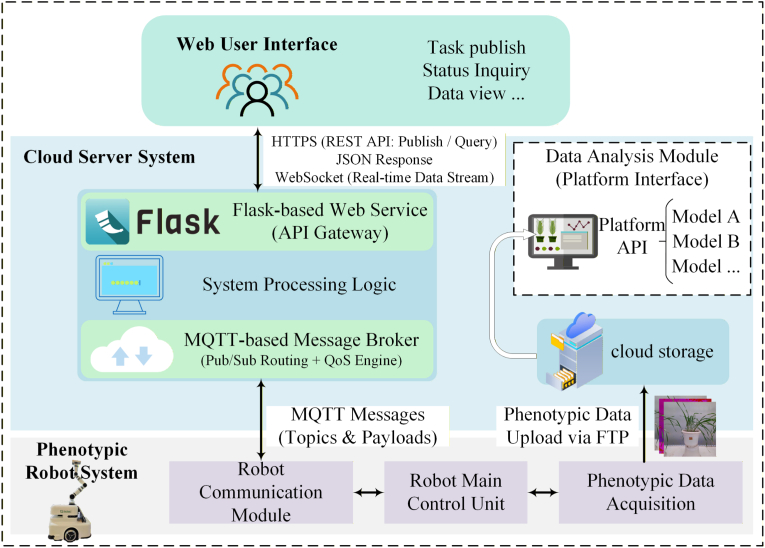
Cloud platform interaction framework diagram.

To enable the application of PhenoRob-P in remotely managed operation and high-throughput task management, this study developed a cloud-based distributed management platform for multiple users and devices. The platform follows a three-level task flow structure, consisting of the user, the cloud service platform, and the robot, and integrates remote task scheduling, status monitoring, and closed-loop data management. Within this workflow, the cloud platform is responsible for task-level coordination and operational supervision, whereas navigation, target localization, pose adjustment, and phenotyping acquisition are autonomously executed by the robot after task delivery. It enables intelligent coordination among multiple robots and comprehensive end-to-end data management (Fig. 5).

The front-end user interface of the cloud management platform was developed using the Vue.js framework. Users can access the platform through a web browser to monitor robot status in real time, including position coordinates, battery level, and task progress. The platform also provides a task management interface, allowing users to configure parameters for phenotyping data collection, such as collection intervals, operational areas, imaging angles, and data types. This design allows users to define and supervise phenotyping missions remotely, while the robot maintains local autonomy during navigation and data acquisition. Once submitted, each task is assigned a unique identifier (UUID) and added to the cloud task queue for distribution and execution.

The back-end system of the cloud management platform is built on the Flask framework and provides task scheduling and status synchronization, supporting asynchronous task distribution and parallel management. Communication between the front-end users and the back end occurs via HTTPS for requests and responses, while real-time or large-volume data is exchanged efficiently through WebSocket. Upon receiving user commands, the back end delivers task information to the robot nodes using the MQTT protocol in a topic-based format. During task execution, the cloud service synchronizes task states and robot feedback, enabling remote supervision and task adjustment without changing the onboard autonomous control process.

On the robot side, each node automatically executes path planning, image acquisition, and pose control based on the received task parameters. Accordingly, the robot-side autonomous navigation mode constitutes the primary execution mode for phenotyping operations, while the cloud platform provides an upper-level management interface for task initiation, progress monitoring, abnormal-condition handling, and data inspection. After data collection, the robot packages the multimodal phenotyping data, including RGB images, point cloud data, and environmental information, and uploads it to the cloud storage node via the FTP protocol. To ensure data integrity and prevent tampering, the upload process incorporates SHA-256 hash verification. Once the upload is successful, the cloud system automatically updates the task status to “data pending analysis” and initiates the data processing workflow. The analysis results are then sent back to the cloud management platform, allowing users to monitor collection progress and data quality, thereby completing a closed-loop information flow from task issuance, data collection, and upload to analysis feedback.

The implementation of this cloud platform substantially reduces the need for on-site human intervention and significantly enhances the intelligent operation of phenotyping robots as well as the efficiency of experimental data handling. By coupling cloud-level task management with robot-side autonomous execution, the system supports scalable phenotyping workflows in which remote operation improves experimental organization, safety supervision, and data traceability, while data acquisition performance remains determined by the onboard navigation, localization, and phenotyping modules. This system establishes a scalable digital infrastructure for phenotyping research in production environments for potted plants, facilitating multi-robot collaboration and the real-time processing of large-scale phenotypic data.

## 3 Experimental validation and results analysis

To assess the operational feasibility of PhenoRob-P in potted-plant facility scenarios, validation experiments were conducted in a glasshouse at Huazhong Agricultural University. The evaluation comprised two complementary components: system-level validation, including high-throughput image acquisition efficiency and LiDAR–vision fusion-based navigation accuracy for pot-level operation, and biological validation, including time-resolved stress phenotyping in wheat and multi-view three-dimensional trait quantification in maize. For clarity, this section is organized as follows. Section 3.1 describes the experimental protocol and evaluation metrics. Section 3.2 presents the results of acquisition efficiency and localization performance. Section 3.3 reports the biological validation results.

### 3.1 Experimental protocol and evaluation metrics

Validation experiments were conducted in 2025 in two adjacent and structurally identical glass greenhouses at Huazhong Agricultural University, Wuhan, China. Each greenhouse measured 12 m × 15 m and contained four rows of container-grown crops. The experimental design was intended to evaluate the robot from two complementary perspectives: engineering performance and biological applicability. The engineering evaluation focused on image acquisition efficiency and target-pot localization accuracy under real greenhouse conditions, whereas the biological evaluation focused on the robot's capability for full-growth-cycle monitoring of wheat phenotypic responses to drought stress and multi-view structural phenotyping.

For the acquisition-efficiency evaluation, two operating modes were defined. In the continuous scanning mode, the robot was equipped with an Azure Kinect depth camera and moved continuously along crop rows at 0.2 m/s while acquiring side-view images at 30 frames per second. In the multi-view fine inspection mode, the camera settings and chassis travel speed were kept unchanged during row traversal. After the robot detected and localized a target pot, the chassis stopped, and the six-degree-of-freedom robotic arm was used to perform spatial pose planning for fine phenotyping. Specifically, the sensor was guided to a set of predefined viewpoints, including a front view, a top view, and left and right side views, to acquire images from multiple observation angles around the plant. Before each image capture at a predefined viewpoint, pose consistency was verified by the dynamic compensation module, and acquisition was triggered only when the angular deviation was below 0.5° and the translational deviation was below 2 mm. During this process, the end-effector scanning speed of the robotic arm was set to 0.2 m/s. This mode was designed to provide more comprehensive geometric and morphological information than continuous side-view scanning while maintaining stable and repeatable sensor poses during data acquisition. The two modes were compared in terms of phenotyping throughput under different observation strategies.

**Fig. 6:**
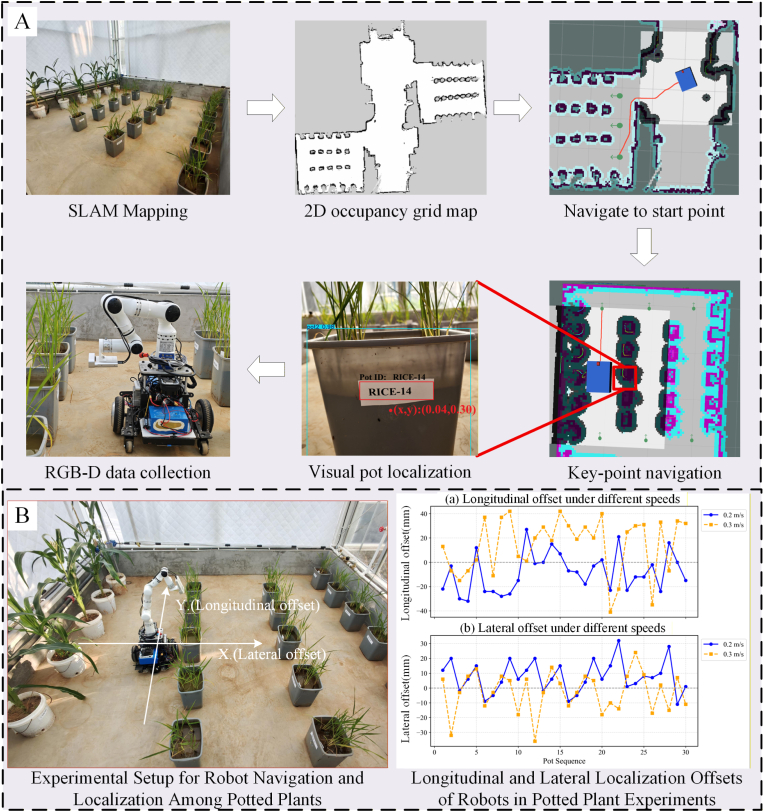
Integrated Navigation Experiment of the Phenotyping Robot. (A)Robot Navigation and Potted Plant Localization in real-world greenhouse environments; (B) Diagram of robot navigation and localization accuracy measurement.

To further evaluate the target-oriented positioning performance of the robotic system in complex greenhouse environments, a localization experiment based on LiDAR–vision fusion navigation was conducted (Fig. 6A). The robot first generated a two-dimensional grid map using SLAM with a map resolution of 3 cm and then navigated along potted-plant rows at two travel speeds, 0.2 m/s and 0.3 m/s. During task execution, the robot operated fully autonomously in row traversal, target detection, target matching, and local alignment, while human involvement was limited to task initialization, safety observation, and post-run measurement. The cloud platform was used for task dispatch and status supervision rather than continuous manual teleoperation; therefore, the reported navigation and acquisition performance reflects robot-side autonomous execution. After the target pot was detected and matched, the robot automatically executed the local alignment procedure and stopped at a predefined acquisition pose in front of the pot. Because close-range phenotyping requires a stable frontal viewing relationship and a repeatable sensor-to-plant distance, the evaluation focused on terminal stopping accuracy rather than full-trajectory SLAM error. Specifically, using the geometric center of the robot as the reference point, the lateral deviation (X) and longitudinal deviation (Y) relative to the desired target-aligned pose were manually measured as the positioning errors (Fig. 6B). Repeated localization experiments were conducted on 30 potted plants under each speed condition, and the results were summarized using the maximum deviation, mean deviation, and standard deviation.

**Fig. 7:**
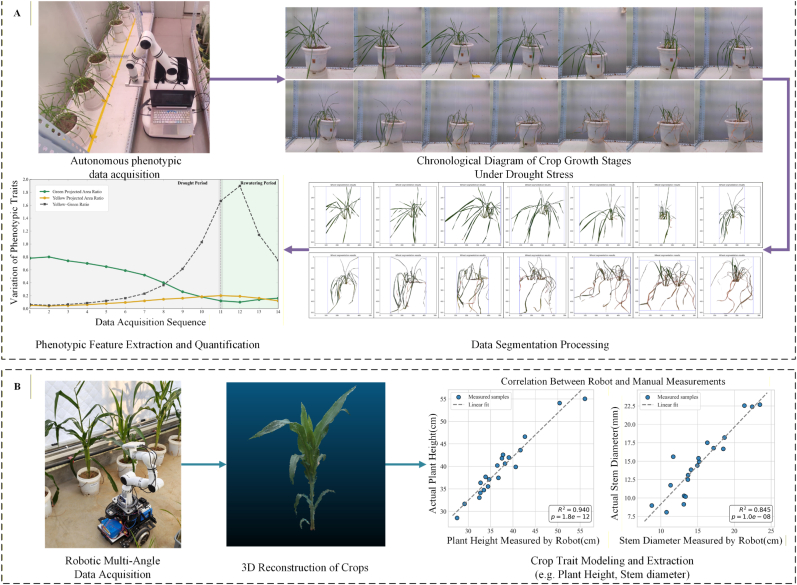
Biological Validation of the Phenotyping Robot. (A) Full-growth-cycle phenotypic tracking of wheat under drought stress; (B) High-precision 3D reconstruction of maize.

For biological validation, two greenhouse experiments were conducted, as shown in Fig. 7. In the wheat drought-stress experiment, 15 wheat varieties were used, with six pots per variety as biological replicates. To ensure comparable initial conditions, each pot was weighed and adjusted to a fresh weight of 600 g before treatment. Drought stress was imposed by withholding irrigation until the soil relative water content decreased to 30%, after which the plants were rewatered on day 14 to restore soil moisture to 80%. The greenhouse was maintained at a 16 h light/8 h dark photoperiod, 22 °C, and 60% relative humidity. During robotic data acquisition, pots were spaced more than 40 cm apart, the minimum robot-to-target distance was maintained above 30 cm, and four viewing angles were used so that more than 90% of each plant remained within the camera field of view. Although slight overlap between leaves from adjacent pots could occur at later growth stages, the target-centered local alignment strategy was used to keep the target plant near the center of the imaging field, thereby reducing the probability that neighboring plants were included in the main region used for trait extraction. Data were collected daily during the first 7 d and every 2 d during the later drought and rewatering stages.

In the maize three-dimensional phenotyping experiment, the six-degree-of-freedom robotic arm autonomously collected multi-view RGB images of individual plants. Five vertical scanning angles were defined around each target plant, and the arm executed a top-down scanning trajectory to capture the layered canopy structure. The acquired images were processed by spatial pose calibration and illumination normalization before three-dimensional reconstruction. Based on the reconstructed plant models, two common structural traits, plant height and stem diameter, were extracted for quantitative analysis. Manual measurements from the same batch of plants were used as ground truth, and the agreement between robotic and manual measurements was evaluated using the coefficient of determination(R^2^).

### 3.2 Phenotyping efficiency and localization performance

The comparison between the two acquisition modes showed a clear trade-off between throughput and observation richness. In the continuous scanning mode, the robot required approximately 6 min to complete full-field data acquisition across the two greenhouses, corresponding to an acquisition efficiency of about 520 pots/h. In contrast, the multi-angle fine inspection mode required about 18 min and achieved an efficiency of about 187 pots/h. These results indicate that the continuous scanning mode is more suitable for rapid large-scale screening, whereas the multi-angle fine inspection mode provides a lower-throughput but more information-rich observation strategy for plants requiring detailed phenotypic characterization. In both modes, the system maintained stable and repeatable data acquisition under greenhouse conditions.

The localization experiment further demonstrated that the LiDAR–vision fusion navigation framework provided stable pot-level positioning performance in a dynamic greenhouse environment. At a travel speed of 0.2 m/s, the longitudinal deviation had a mean of 15.6 mm, a standard deviation of 9.8 mm, and a maximum deviation of 32 mm, whereas the lateral deviation had a mean of 10.5 mm, a standard deviation of 7.8 mm, and a maximum deviation of 32 mm. When the speed was increased to 0.3 m/s, the longitudinal deviation increased to a mean of 23.8 mm with a standard deviation of 12.6 mm and a maximum deviation of 42 mm, while the lateral deviation remained relatively stable, with a mean of 11.1 mm, a standard deviation of 8.1 mm, and a maximum deviation of 36 mm. Approximately 87% of positioning errors in both directions were within ±30 mm. These results indicate that increasing travel speed mainly affected longitudinal stopping accuracy, whereas lateral deviation remained well controlled, suggesting that the vision-assisted correction strategy effectively constrained sideways drift. Taken together, the results confirm that the proposed navigation framework can satisfy the positioning accuracy requirements of high-throughput phenotyping in constrained greenhouse rows. Detailed statistics are summarized in Table 2.

**Table 2:** Statistical results of navigation and positioning accuracy for agricultural robots in controlled environments.

Evaluation Metric	0.2 m/s		0.3 m/s	
	Lateral Deviation (X)	Longitudinal Deviation (Y)	Lateral Deviation (X)	Longitudinal Deviation (Y)
Maximum Deviation (mm)	32	32	36	42
Mean Deviation (mm)	10.5	15.6	11.1	23.8
Standard Deviation (mm)	7.8	9.8	8.1	12.6

### 3.3 Biological validation results

In the wheat drought-stress experiment, the robotic platform enabled non-contact phenotypic tracking of plant responses across the full crop growth cycle, including both drought progression and post-stress recovery. The acquired images were processed by background segmentation, which achieved an accuracy of 98% and a mean intersection over union of 85%, providing the basis for subsequent trait extraction. Using a representative wheat variety as an example, temporal changes in the green area ratio (GAR), yellow area ratio (YAR), and yellow-to-green ratio (YGR) reflected the progression of drought stress. During the early drought stage (days 1–3), GAR remained around 0.80, YAR remained around 0.05, and YGR remained around 0.065, indicating that leaf color remained largely normal. From days 4 to 8, GAR decreased markedly while YAR increased, indicating progressive leaf yellowing with increasing drought stress. From days 10 to 14, GAR continued to decline and YAR continued to rise, reflecting further aggravation of stress symptoms. After rewatering on day 14, GAR showed partial recovery and YAR decreased slightly, indicating limited morphological recovery, although some drought-induced yellowing remained irreversible. These temporal trends indicate that the system was able to capture phenotypic changes associated with drought onset, progression, and partial recovery in greenhouse-grown wheat.

In the maize three-dimensional phenotyping experiment, the robot achieved stable multi-view image acquisition and reconstructed detailed three-dimensional plant architecture under greenhouse conditions. The reconstructed model preserved the layered canopy structure and clear internode morphology, enabling automatic extraction of structural traits including plant height and stem diameter. Compared with manual measurements, the robotic estimates showed strong agreement, with R^2^ = 0.940 for plant height and R^2^ = 0.845 for stem diameter. These results indicate that the robot can recover major architectural traits of maize and provide structurally consistent data for subsequent growth analysis and architectural phenotyping.

## 4 Discussion

The results indicate that PhenoRob-P fills a specific but practically important niche in the current phenotyping-platform landscape: repeatable plant-specific phenotyping in structured potted production environments where narrow aisles, GNSS denial, and the need for target-level data traceability are more critical than large-area coverage alone. In this sense, the positioning accuracy achieved here should not be interpreted merely as a navigation outcome, but as an enabling condition for phenotypic repeatability. For phenotypic tracking across the full crop growth cycle, small errors in stopping pose can propagate into variations in view geometry, image scale, self-occlusion patterns, and illumination conditions, thereby reducing the comparability of phenotypic measurements across time points even when the downstream trait-extraction algorithm remains unchanged. Environmental effects should therefore be interpreted in a module-dependent manner. For LiDAR-based row navigation, performance is mainly affected by the geometric observability of greenhouse structures, reflective surfaces, and dynamic obstacles, whereas vision-based modules are more sensitive to illumination variation, plant–background contrast, and target appearance. The present results therefore support an application-oriented argument: in potted greenhouse phenotyping, navigation accuracy matters primarily because it stabilizes the sensing context for repeated biological observation, rather than because it improves locomotion performance in isolation [11,14,17].

A second point emerging from the results is that the main contribution of PhenoRob-P lies in workflow integration rather than in any single standalone robotics component. Existing platforms already demonstrate the effectiveness of mobile phenotyping in both field and greenhouse settings, but their operational priorities are different. Field robots such as PhenoRob-F and TerraSentia emphasize traversal efficiency, large-scale coverage, and trait acquisition under open or under-canopy field conditions, where throughput and robustness across heterogeneous plots are central [[24], [25], [26]]. Greenhouse platforms such as PATHoBot and G-ROBOT illustrate the value of robotic crop inspection in protected environments, but they are generally oriented toward particular tasks, crop types, or infrastructure assumptions [27,28]. By contrast, the present system targets a different operational bottleneck: maintaining reliable pot-level identity association and repeatable close-range multi-view acquisition in a GNSS-denied, narrow-row facility environment. This distinction is important, because in breeding-oriented potted experiments the principal challenge is often not merely robot traversal, but the reliable association of phenotypic data with the correct individual over time under pot rearrangement and growth-driven changes in plant appearance and structure. From that perspective, the combination of LiDAR-guided row traversal, vision-based target confirmation, local alignment, and arm-based adaptive sensing constitutes a task-complete phenotyping workflow rather than a simple aggregation of standard robot modules [11,14,28].

The engineering results also help explain the biological outcomes. The performance differences observed across the two application scenarios reflect a practical trade-off in greenhouse phenotyping between acquisition efficiency and structural completeness. In the present study, both modes relied on multi-view image acquisition rather than simple pass-through inspection, but they differed substantially in acquisition flexibility, viewpoint richness, and operational cost. The wheat experiment adopted a relatively standardized four-angle acquisition strategy, enabling rapid and repeatable plant-level observation at high throughput. This configuration was therefore well suited for full-growth-cycle phenotypic tracking tasks, in which consistent visual records and temporal comparison at the population level are primary objectives. By contrast, the maize experiment employed a robotic-arm-based multi-angle scanning strategy with higher degrees of freedom, allowing the sensor to acquire more detailed views of the plant from multiple spatial positions. Although this mode reduced operational throughput, it provided richer geometric support for downstream three-dimensional reconstruction and structural trait analysis. The relatively strong agreement obtained for plant height and stem diameter suggests that the contribution of the robotic arm is not simply to increase the number of images acquired, but to improve the spatial diversity, geometric completeness, and viewpoint controllability of the observations, thereby increasing the utility of the collected data for reconstruction-based phenotyping. This interpretation is consistent with recent studies showing that the quality of 3D phenotyping depends not only on reconstruction algorithms themselves but also on acquisition geometry, viewpoint planning, and image redundancy [9,38,39]. Accordingly, the present results support a task-oriented phenotyping framework in greenhouse research, in which the platform can perform continuous uninterrupted scanning when rapid coverage is required, can also operate in an efficient multi-angle acquisition mode for routine temporal monitoring, and can further switch to a higher-flexibility fine-acquisition mode when structure-sensitive traits require richer geometric support.

Its validation was conducted in a greenhouse environment where pots were arranged with relatively sufficient spacing and target plants were generally accessible. This experimental setup was adopted not because the system is restricted to sparse layouts, but because a relatively clear observation scene facilitates more intuitive visualization of phenotypic differences and temporal changes across successive measurements. In early growth stages, the scene is relatively simple, whereas continued plant development gradually increases occlusion, structural complexity, and the difficulty of repeatable close-range observation. For vision-based phenotyping, illumination variation, low plant–background contrast, and large changes in container appearance may influence pot detection, identifier recognition, plant segmentation, and color-index extraction. In the current workflow, RGB-D sensing, target-centered local alignment, multi-view acquisition, and background segmentation help mitigate these effects under real greenhouse conditions. For broader deployment, the visual models can be further fine-tuned with representative images from target environments to improve adaptability across diverse lighting, background, and container-appearance conditions. Within this context, the robot is able to operate under potted-plant arrangements that are not fully standardized, and the proposed target association and local alignment strategy improves adaptability to practical facility scenarios in which pot placement may be irregular or adjusted over time. At the same time, important limitations remain in denser canopies with severe leaf overlap or in multilayer cultivation facilities commonly encountered in commercial greenhouse production, where the difficulty of target separation, safe access, and stable close-range observation would increase substantially. Such overlap may introduce non-target plant regions into trait extraction, particularly for projected-area, color-ratio, and reconstruction-based measurements. Although target-centered acquisition and segmentation reduce moderate interference, severe structural mixing remains a subject for future work. In particular, while the 2D LiDAR used in this study is adequate for global localization and row-level motion control, it does not by itself represent the full three-dimensional complexity of crowded facility scenes [37]. Likewise, the current platform is not yet configured for efficient phenotyping in multilayer cropping environments. However, this limitation is mainly related to the present hardware configuration rather than the overall task framework, and could be alleviated in future work through the integration of a lifting module to extend vertical reach and support phenotyping under denser and more vertically heterogeneous crop conditions. In addition, the current workflow still relies on pot identifier recognition for stable target-to-data association. This design remains practical for many breeding and experimental workflows, where pot-level identity management is still required, but it also indicates that the system has not yet achieved fully marker-free long-term tracking.

These limitations also define the most meaningful directions for future work. One priority is to move from identifier-dependent target association toward marker-free plant or pot re-identification using multimodal cues from geometry, texture, topology, and temporal context, which would improve scalability in breeding programs and reduce manual preparation. A second priority is to improve perception and active sensing in more cluttered greenhouse scenes by introducing richer 3D perception, uncertainty-aware target localization, and adaptive viewpoint planning, so that the system can handle denser canopies and more variable crop architectures. In parallel, continued adaptation of the vision models with data from different greenhouse layouts, lighting conditions, backgrounds, and container types would further improve cross-scenario robustness without changing the overall system architecture. A third direction is to strengthen system-level autonomy beyond motion execution by incorporating online data-quality assessment, task rescheduling, and coordinated multi-robot operation for larger facilities. In this sense, the significance of PhenoRob-P does not lie in claiming a complete solution to greenhouse phenotyping robotics, but in providing an experimentally validated platform on which marker-free identity management, robust active perception, and scalable phenotyping workflows can be further investigated in realistic facility conditions [9,11,39,40].

## 5 Conclusion

This study developed PhenoRob-P, an autonomous robotic phenotyping system for potted plant production environments, with the aim of enabling repeatable, plant-specific, and time-resolved data acquisition under narrow-aisle and GNSS-denied greenhouse conditions. Rather than introducing a completely new generic robotics algorithm, the main contribution of this work lies in establishing a task-oriented phenotyping framework that integrates compact mobile operation, LiDAR–vision collaborative localization, pot-level target association and local alignment, robotic-arm-based adaptive multi-view acquisition, and cloud-enabled task and data management into a unified workflow.

Within the context of controlled-environment agriculture, this integrated design provides a practical solution for bridging the gap between fixed infrastructure-based phenotyping systems and conventional mobile platforms. It combines the deployment flexibility of autonomous ground robots with the repeatable sensor-to-plant relationship required for pot-level phenotypic analysis, thereby supporting full-growth-cycle phenotypic tracking and monitoring, structural trait quantification, and breeding-oriented data collection in greenhouse production scenarios.

The current platform is most suitable for structured or semi-structured greenhouse environments with relatively accessible target plants and sufficient working space for robot maneuvering and close-range sensing. Its application in dense commercial canopies, multilayer cultivation systems, and highly heterogeneous production environments remains limited, and the current workflow still depends on identifier-based target association for stable target-to-data linkage. In addition, practical deployment still involves challenges related to hardware cost, calibration consistency, and long-term system maintenance.

Future research will focus on marker-free plant or pot re-identification, more robust perception and viewpoint planning in cluttered greenhouse scenes, tighter integration of online task planning with data-quality-aware acquisition, and more economical modular configurations for broader deployment. Further efforts will also extend toward embodied-AI-enabled agricultural robotics and multi-robot coordination for collaborative phenotyping, so as to improve system-level autonomy, adaptability, and operational scalability in complex controlled-environment production systems.

## CRediT authorship contribution statement

Y.S. and Y.H. designed the robot, analyzed the data, and wrote the manuscript. Y.F. and Z.L. performed the field experiment. R. Z. also performed the experiments and analyzed the data. W.Y. and P.S. supervised the project.

## Declaration of competing interest

The authors declare the following financial interests/personal relationships which may be considered as potential competing interests: Given his role as Associate Editor, Wanneng Yang had no involvement in the peer review of this article and had no access to information regarding its peer review. Full responsibility for the editorial process for this article was delegated to another journal editor.

## Data Availability

The source code and sample datasets supporting the findings of this study are openly available at the following GitHub repository: https://github.com/Sunniersy/PhenoRob-P. All other data and research materials that support this study are available from the corresponding author upon reasonable request.
